# Antenatal Care Attendance and Factors Influenced Birth Weight of Babies Born between June 2017 and May 2018 in the Wa East District, Ghana

**DOI:** 10.1155/2020/1653076

**Published:** 2020-07-19

**Authors:** Prince Kubi Appiah, Mohammed Bukari, Simon Nidoolah Yiri-Erong, Kwabena Owusu, George Borogyante Atanga, Stephen Nimirkpen, Blaise Bagyliku Kuubabongnaa, Martin Adjuik

**Affiliations:** ^1^Department of Family and Community Health, School of Public Health, University of Health and Allied Sciences, Ho, Ghana; ^2^Department of Medical Law and Ethics, Asian Institute for Bioethics and Health Law, College of Medicine, Yonsei University, Seoul, Republic of Korea; ^3^Health Information Unit, District Health Administration, Ghana Health Service, Wa East District, Upper West Region, Ghana; ^4^Department of Public Health and Community Medicine, Institute of Medicine, University of Gothenburg, Sweden; ^5^Health Information Unit, District Health Administration, Ghana Health Service, Wa West District, Upper West Region, Ghana; ^6^Health Information Unit, District Health Administration, Ghana Health Service, Sissaala West District, Upper West Region, Ghana; ^7^Health Information Unit, Municipal Health Administration, Ghana Health Service, Lawra Municipal, Upper West Region, Ghana; ^8^Department of Epidemiology and Biostatistics, School of Public Health, University of Health and Allied Sciences, Ho, Ghana

## Abstract

**Background:**

In sub-Saharan Africa, there is high coverage of the recommended four or more times antenatal care (ANC) visits during pregnancy without complications; notwithstanding this achievement, the negative birth outcomes related to childbirth such as low birth weights and stillbirths are still high despite the increased access to antenatal services. Hence, the study assessed the association between antenatal attendance and birth weight in the Wa East District.

**Method:**

The cross-sectional study design was used with a semistructured questionnaire to collect data from mothers who delivered within a one-year period through a review of antenatal and birth records from health facilities where the women delivered and interviewed. The chi-squared test and univariate and multivariate logistic regression were performed to establish the association between normal birth weight and ANC services the woman received and other predictor variables, and *p* value < 0.05 was considered a significant association between dependent and independent variables.

**Result:**

The study involved 233 women. About 62.2% attended ANC clinics 4+ times before giving birth, 70.0% did not received the minimum ANC services required for every pregnant woman, 0.9% of pregnancies resulted in stillbirth, and 24.5% of babies born had a birth weight < 2.5 kg. Women marital status (legally married) [AOR: 2.05, 95% CI: 1.33-6.89, *p* = 0.044], religion (Islam) [AOR: 0.33, 95% CI: 0.08-0.39, *p* = 0.013], and educational level (SHS/tertiary) [AOR: 4.27, 95% CI: 0.08-0.88, *p* = 0.031] were the background characteristics associated with normal birth weight (2.5-40 kg). Also, women who had their urine tested at the ANC clinics [AOR: 6.59, 95% CI: 8.48–15.07, *p* < 0.001] and women who received a long-lasting insecticide-treated net [AOR: 2.17, 95% CI: 0.03-0.92, *p* = 0.039] from the ANC clinic were associated with normal birth weight.

**Conclusion:**

Notwithstanding the benefits of antenatal care services, only 62.2% of pregnant women attended 4 or more ANC visits before giving birth, while 70% did not received the services they need. These might have influence the 24.5% of babies born with a low birth weight. Therefore, there is a need for special attention from all stakeholders to reverse the trend.

## 1. Introduction

Antenatal care (ANC) is a fundamental component of routine maternal and child health services and provides opportunities for many different services to be offered to pregnant mothers with the aim of screening and detecting danger signs early and providing the necessary timely interventions. The goal of antenatal care is to reduce maternal and child mortality and morbidity; for example, the roll-out of antimalarial drugs and antiretroviral therapy for maternal HIV/AIDS are services offered at antenatal care sessions to reduce maternal and child morbidity and mortality [1]. Therefore, frequent visits to the ANC clinic are crucial in realizing the full benefits of ANC services. Antenatal services such as tetanus toxoid-diphtheria vaccination, IPTp for malaria control, and HIV prevention of mother-to-child transmission (PMTCT) depend on several visits and the trimester in which they occur to be effective [2].

Pregnant women stand a greater risk of losing their lives or their babies if proper care and attention are not given to them during antenatal care until delivery. Globally, in 2015, the maternal mortality ratio was estimated to be around 216 per 100,000 live births and occurring mostly in low-resource settings [3] due to pregnancy-related causes [4] while skilled attendance at birth coverage in 2013 was estimated to have reached 73%; however, more than 40% of births in WHO Africa Region were not attended to by a skilled health personnel [5]. Also, globally, an estimated 5.9 million children in less than 5 years lost their lives in 2015, with neonatal and under-five mortality rates of 19 per 1,000 live births and 42.5 per 1,000 live births, respectively, occurring; however, the highest mortality occurred in sub-Saharan Africa, where antenatal care (4+ visits) in 2015 was 55% [6]. It is also known that good quality of care at childbirth can produce a triple effect on any investment made, saves mothers and newborns, and prevents stillbirths and low birth weights. Therefore, the provision of effective care for all women and babies during birth in health facilities could prevent about 113,000 maternal deaths; 531,000 stillbirths; and 1.3 million neonatal deaths that occurred annually by 2020 [6]. Yet, about half of the under-five deaths are caused by malaria, tetanus, measles, sepsis, pneumonia, and AIDS which are directly associated with the lack or insufficient antenatal care services [7].

In Ghana, the maternal mortality ratio is estimated at 310/100,000 live births, while neonatal and infant mortality rates were 25/1,000 and 37/1,000 pregnancies [8], respectively. Also, the Ghana Demographic and Health Survey estimates ANC coverage at 97.3%, ANC 4+ visits at 82.9%, and low birth weight at 9.5% [9]. In particular, the Wa East District has seen some improvement over the years in ANC coverage from 84.6% in 2017 to 85.8% in 2019, while ANC 4+ visits increased from 70.7% in 2017 to 71.9% in 2019. With the consistent increase of ANC coverage and significant coverage of the 4th visit, it was expected that the negative birth outcomes such as stillbirths and low birth weights would have been reduced to the minimum levels. But, the district recorded an increase in the low birth weight rate from 6.2% in 2017 to 7.5% in 2019, which could be a contributory factor to neonatal deaths and increased in stillbirths from 5 per 1,000 pregnancies in 2017 to 15 per 1,000 pregnancies in 2019 [10]. Although the district is yet to fall above the national ANC coverage (80%) and ANC 4+ visits (78%) targets, the increase in stillbirth and low birth weight rates amidst the increased ANC coverage and ANC 4+ visits suggests an investigation; hence, the study examined the association between antenatal care attendance and birth outcomes among mothers who delivered from June 2017 to May 2018 in the Wa East District.

## 2. Materials and Methods

### 2.1. Study Site

The Wa East District is a complete rural district with 145 communities. Currently, the district has an estimated population of about 87,182 with a 3.9% fertility rate. There are 9 health centres, a health post/clinic, and 28 functional community-based health planning and service (CHPS) facilities in the district rendering health services to the people. Although there are no hospitals and polyclinics in the district, the health centres there possess the needed resources and the potentials to render the basic emergency obstetrics and newborn care (EmONC) services to whoever needs these services [10].

### 2.2. Study Population

The study population includes women aged 15 to 45 years, who attended ANC clinics and delivered in health facilities in the district between June 2017 and May 2018. However, women who went through a caesarean section before delivery were excluded from the study because a study has shown that babies delivered through a caesarean section may gain more weight due to the fluids given to the mother before and during surgery [11]. Also, women with missing ANC and delivery data from health facility records, health professionals, and foreign nationals were excluded from the study.

### 2.3. Study Design

A descriptive cross-sectional design was employed to collect the study data at one point in time. The study involved reviewing individual records of women who gave birth at a health facility between June 2017 and May 2018 and interviewed the women for additional information. Variables that data were collected in the facility include parity, number of ANC visits made before delivery, birth weight of the child, still/live birth, and a minimum amount of ANC services received by mothers before delivery. Data collected from the mothers directly include the mothers' demographic data and counselling information given to mothers during ANC visits. Mothers' ANC booklets were referred to as where necessary to ascertain the needed information.

### 2.4. Sample Size

The sample size of two hundred and thirty-three (233) participants involved in the study was determined using the Cochran formulated formula for cross-sectional population-based studies: *n* = *z*^2^ × *p*(*q*)/*d*^2^ [12], where *n* was the sample size to be determined, *z* was the *z*-score of the area under the standard normal curve corresponding to the desired confidence level of 95% (1.96), *p* was the prevalence of low birth weight of 16.5% (0.165) in the population [13], *q* was 1-*p*, and *d* was the 5% (0.005) acceptable margin of error. Considering a 10% nonresponse rate for the 212 participants, the sample size, finally became 233 participants. This was to ensure that the estimated prevalence fell within ±5% of the true population coverage, with a probability of 95%.

### 2.5. Sampling Method

Nine health centres were providing basic emergency obstetrics and newborn care (EmONC) services in the district. Hence, we purposively selected all the nine health centres for the study, and a probability proportionate to the size sampling method was used to allocate the sample size to each health centre based on the total deliveries conducted between 1^st^ June 2017 and 31^st^ May 2018. After getting the sample size for the health centres, the number of deliveries registered in each health centre's delivery register became a sample frame, and a systematic sampling method was used to select the number of women allocated to the health centre. The selected women were traced to their homes using the contact information in their health records.

### 2.6. Data Collection Tools and Procedure

A semistructured questionnaire was used for data collection, and pretesting of the tool was done using five (5) women who were not part of the study participants to ensure that it addressed all the objectives without ambiguity. Four nurses who were familiar with ANC activities and services were recruited from outside the study area, and were trained to collect data for the study, and were persons who understood the local dialect and could explain the questionnaire to the understanding of the study participants using the three main languages in the area, namely, Dagaare, Sisaali, and Chakali. The participants were interviewed, and their ANC and delivery records were reviewed. Information collected includes age, educational level, religion, parity, gestational age at first ANC visit, number of ANC visits, child's birth weight, livebirth, stillbirth, and counselling during ANC visits.

### 2.7. Data Analysis

The analytical software STATA version 13.1 was used for data analysis, and frequencies and proportions were used to report on ANC attendance, minimum ANC services received, and other variables. The minimum ANC service received was determined using the following fourteen services provided to pregnant women during an antenatal care visit: measured weight, height, blood pressure, and hemoglobin level; assessed gestational age; performed urine, syphilis, and PMTCT tests; gave iron-folic acid supplementation, tetanus-diphtheria injection, sulphadoxine-pyrimethamine (SP) tablets, and LLIN; and counselled on nutrition and family planning services. Mothers who received 13 or less of these services were classified as getting below the minimum requirements. The chi-squared and logistics regression tests were performed to determine the association between independent and dependent variables. The *p* value < 0.05 was considered statistically significant.

## 3. Results

### 3.1. Background Characteristics of Participants

A total of 233 women who attended ANC clinics and delivered in health facilities in the district were interviewed and health records reviewed with more than half 148 (63.5%) of them being within the ages of 20 to 34 years and a mean age of 27 years ± 7.0 standard deviation. Majority 215 (92.3%) of them were legally married and as high as 113 (48.5%) of the women have no formal education while only 17 (7.3%) have secondary and tertiary education. More than half 149 (64.0%) of them were farmers, and 53 (22.7%) were unemployed. Islamic religion 167 (71.7%) was the major form of religious expression in the district. About 56 (24.0%) of the women had delivered four or more children before the current child, while 99 (42.5%) and 78 (33.5%) of the women had delivered 1 and 2-3 times before the current child they have (Table 1).

### 3.2. Antenatal Services Women Received

About 145 (62.2%) of the women attended four or more ANC clinics for care while 12 (5.2%) went to ANC clinics only once. All the women had their weight and height measured, and blood pressure checked. Also, about 229 (98.3%) received iron-folic supplementation of which 84 (36.7%) and 100 (43.7%) had it four and five or more times, respectively, 161 (69.1%) were given sulphadoxine-pyrimethamine (SP) tablets out of which 107 (66.5%) got it twice, 202 (86.7) got tetanus-diphtheria vaccination, and 171 (73.4%) received a long-lasting insecticide-treated net (LLITN). Again, 230 (98.7%) were tested against PMTCT, 204 (87.6%) had their urine tested and 228 (97.9%) tested for syphilis, and 222 (95.3%) had their hemoglobin levels checked, while 231 (99.1%) had gestational age checked, and 223 (95.7%) and 230 (98.7%) received counselling on family planning and nutrition, respectively. Generally, only 30.0% of the women received all the 14 minimum services required (Table 2).

### 3.3. Pregnancy Outcome and Birth Weight

**Figure 1:**
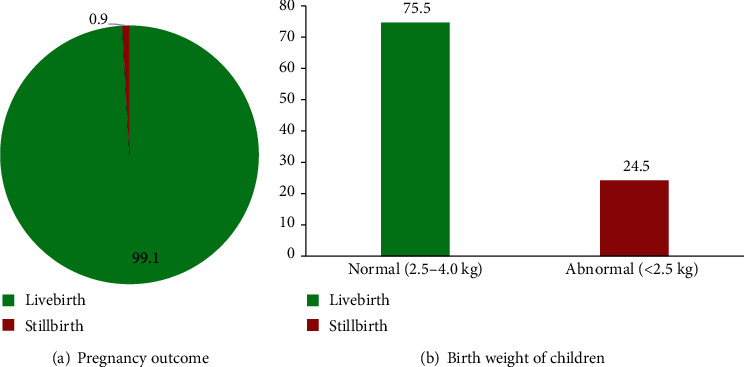
(a) Pregnancy outcome. (b) Birth weight of children.

About 2 (0.9%) out of the 233 pregnancies resulted in stillbirth (Figure 1(a)), while 57 (24.5%) of the babies born had birth weight below 2.5 kg (abnormal) (Figure 1(b)).

### 3.4. Associations between Background Characteristics and Normal Birth Weight

The chi-squared analysis showed significant associations between birth weight and marital status (*p* = 0.04), religion (*p* = 0.016), and educational level (*p* = 0.003) of the women. To further confirm the associations, bivariate logistic analysis was performed, and it was confirmed and showed associations between normal birth weight and legally married women [COR: 2.71, 95% CI: 1.01-7.24, *p* = 0.047], women who were Muslims [COR: 0.39, 95% CI: 0.18-0.85, *p* = 0.018], and women who have attained secondary/tertiary education [COR: 3.15, 95% CI: 0.04-0.61, *p* = 0.008]. The associations indicated that married women were 2.7 times more likely to give birth to children with a normal birth weight than women who were in cohabitation relationships, women who were Muslims were 61% less likely to deliver babies with a normal birth weight than those who were Christians, while women with secondary/tertiary education were about 3.2 times more likely to give birth to children with a normal birth weight than women with no education.

Furthermore, when variables that were having associations with normal birth weight from the bivariate analysis were tested for confounding effects using multiple logistic regression analysis, it was further confirmed that women who are legally married [AOR: 2.05, 95% CI: 1.33-6.89, *p* = 0.044], women who were Muslims [AOR: 0.33, 95% CI: 0.08-0.39, *p* = 0.013], and women who have attained secondary/tertiary education [AOR: 4.27, 95% CI: 0.08-0.88, *p* = 0.031] were associated with normal birth weight. This also indicated that legally married women were about 2.1 times more likely to give birth to children with a normal birth weight than women who are in cohabitation relationships, women who were Muslims were 67% less likely to deliver babies with a normal birth weight than those who were Christians, while women who have attained secondary/tertiary education were 4.3 times more likely to give birth to babies with a normal birth weight than women who had no education (Table 3).

### 3.5. Associations between Antenatal Services the Woman Received and Normal Birth Weight

Again, chi-squared analysis showed significant associations between the birth weight and checking of hemoglobin levels (*p* = 0.002), urine test (*p* = 0.000), times sulphadoxine-pyrimethamine (SP) tablets received (*p* = 0.018), syphilis test (*p* = 0.003), LLITN distribution (*p* = 0.002), counsel on FP (*p* = 0.008), counsel on nutrition (*p* = 0.013), and birth outcome (*p* = 0.013).

To further confirmed the associations, a bivariate logistic analysis was performed, and this confirmed and showed associations between a normal birth weight and women who had their hemoglobin levels tested [COR: 6.02, 95% CI: 1.69-21.39, *p* = 0.006], women who had their urine tested [COR: 15.18, 95% CI: 6.23-8.28, *p* < 0.001], women who received SP tablets 2 times [COR: 1.37, 95% CI: 0.16-0.86, *p* = 0.021], women who were tested for syphilis [COR: 3.21, 95% CI: 1.45-2.73, *p* = 0.022], women who received LLITN [COR: 3.26, 95% CI: 0.11-0.65, *p* = 0.004], and women who were counselled on FP [COR: 5.06, 95% CI: 1.37-8.62, *p* = 0.015]. The associations indicated that women who had their hemoglobin levels checked were 6 times more likely to give birth to children with a normal birth weight than women whose hemoglobin levels were not tested, women who had their urine tested were 15.2 times more likely to deliver babies with a normal birth weight than those without urine test, women who received SP tablets 2 times were about 1.4 times more likely to give birth to children with a normal birth weight than women who received SP once, women who were tested for syphilis were 3.2 times more likely to give birth to babies with a normal birth weight than those who were not tested, and women who received LLITN were also 3.3 times more likely to deliver babies with a normal birth weight than those who were not given, while women who were counseled on FP were 5.1 times more likely to deliver children with a normal birth weight than those who were not counseled.

Additionally, when variables that were having associations with normal birth weight from the bivariate analysis were tested for confounding effects using multiple logistic regression analysis, it was confirmed that women who had their urine tested [AOR: 6.59, 95% CI: 8.48–15.07, *p* < 0.001] and women who received LLITN [AOR: 2.17, 95% CI: 0.03-0.92, *p* = 0.039] were associated with normal birth weight. This indicated that women who had their urine tested and women who received LLITN were 6.6 and 2.2 times more likely to give birth to babies with a normal birth weight than women whose urine were not tested and those who did not receive LLITN, respectively (Table 4).

## 4. Discussion

Almost half (48.5%) of the mothers in Wa East District never went to school. Meanwhile, decreasing levels of education has been linked to lower birth weight [14]. Again, maternal educational level is known to influence child health as well as reducing unhealthy behaviours, including smoking [15, 16]. Mothers who are employed and work characteristics have been associated with reduced low birth weight [17], yet the present study showed that 22.7% of mothers are unemployed. Another study in Nigeria also indicated that higher maternal education and occupation were associated with higher mean birth weights and showed that low birth weight was higher among mothers in the lower social class compared with mothers in the upper social class [18]. About 7.7% of mothers in the current study were involved in cohabiting relationships. Meanwhile, a study has shown that mothers who were not married are associated with an increased risk of low birth weight [19]. Though a study has shown that advancing in maternal age enhances birth weight by influencing foetal growth, 17.6% of mothers in the present study were below 20 years of age [20].

Globally, pregnant women are to make at least four or more antenatal care visits from skilled personnel before giving birth [21]. However, only 62.2% of mothers in the present study made four or more ANC visits, which is far below the 80% target set for all districts to achieve and Ghana's demographics and health survey [9, 22]. Contrary, the Nigeria and Eastern Sudan studies reported lower proportions (54% and 11%, respectively) [23, 24]. Admittedly, a cross-sectional study in Kumasi, Ghana, indicated the lack of ANC attendance to the fact that mothers were not sick and so there was no need to visit ANC clinics or hospitals, as well as cultural beliefs [25]. These could also be the reasons why mothers in the Wa East District were not attending ANC clinics, although why mothers were not attending ANC clinics were not captured in the present study. The minimum ANC package in the study refers to the 14 services outlined in Table 2 in Results of the paper which are provided for pregnant mothers at the ANC clinic. However, the study revealed that only 30% of the mothers received all ANC services required. Partly, the pregnant women who were unable to attend the recommended four or more visits could not have received all services. Again, in a rural district such as Wa East, where there are no laboratory facilities, services such as urine test and hemoglobin checks are not readily offered. Therefore, pregnant women are usually requested to visit another district for these services to be rendered. As a result, some mothers find it inconvenient and may not adhere to the referral and hence did not receive all the services that every pregnant woman is required. Also, shortages of logistics, such as iron-folic acid tablets, long-lasting insecticide-treated nets, sulphadoxine-pyrimethamine tablets, and HIV and syphilis test kits, mean that mothers who attended ANC clinics at the times of shortages were denied such services. Hence, most pregnant women did not receive ANC services required. Nonetheless, a comparative cross-sectional study in northern Ghana made a similar finding and indicated that only 34.6% of mothers received adequate ANC care services [26]. Again, a study conducted in Bangladesh reported a lower proportion (22%) of mothers who received all the prescribed ANC services [27]. The Bangladesh study also revealed that mothers who received the minimum ANC service were about 2.4 times more likely to give birth to children with a normal birth weight (2.5–4.0 kg) compared with those who received fewer services. This finding was not surprising, because mothers who attend ANC sessions and receive all services required and put the pieces of advice into practice have more chance of delivering healthy babies [28].

Approximately 24.5% of babies born between June 2017 and May 2018 were below the normal birth weight (<2.5 kg). In contrary, the Nigerian studies and the Ethiopian study reported prevalence (6.3%, 10.2%, and 17.1%) of a low birth weight, which are lower than what the present study reported [29–31]. Meanwhile, low-birth-weight children are known to suffer from lack of proper physical growth and mental development, are susceptible to infections and physical handicaps, and are prone to emotional disorders [32]. About 1% of pregnancies in the Wa East District resulted in a stillbirth. This disagrees with a study carried out in the Hohoe Municipality in Ghana, which reported a high prevalence of stillbirth [33] and the study among the ethnically diverse population in the United Kingdom [34].

Religion was found to be associated with birth weight in the present study and agrees with a study in India [35], and in Africa, the tradition of selection and preference of nutritionally poor food items during pregnancy is deep-rooted in communities and may be due to religious practices that affect maternal healthcare-seeking behaviour and nutrition [36]. Again, another study has shown that maternal religious attendance has a protective effect against low birth weight [37]. Maternal marital status was also found to be associated with normal birth weight in the present study and agrees with the Nigerian studies [29, 30]. However, mothers who are not married are known to have a higher risk of delivering low-birth-weight children [19]. The present study also indicated that mothers who have attained higher education were more likely to give birth to babies with a normal birth weight compared with mothers who have attained lower education or did not go to school. This educational finding concurs with Tayie and Lartey's study, which reported that pregnant women with higher educational attainment turn to seek early care and that those who seek early care are likely to give birth to children with a normal birth weight (2.5-4.0 kg) than women with no education [38]. Meanwhile, maternal education has an influence on negative health behaviours, which in turn impact child health outcomes [15, 16].

Mothers who had their urine tested were found to be associated with given birth to a child with a normal birth weight, and a study has shown that mothers who had their urinary metabolites measured at the end of the first trimester are associated with an increased risk of negative birth outcomes [39]. Also, mothers who were given LLITN to help protect them from mosquitoes' bite and prevent malaria were significantly associated with a normal birth weight, with a study indicating reduce birth weight among mothers who has malaria during pregnancy [40]. These services are part of the minimum services all pregnant women were supposed to receive from ANC facilities visited. A study has shown that mothers who did not go for antenatal care and for that reason could not receive services required were associated with low birth weight and other birth outcomes [41]. All efforts should be instituted to ensure pregnant mothers receive all required services because these services are essential to ensure both the mother and child's life are saved.

## 5. Conclusion

Notwithstanding the benefits of antenatal care services, only 62.2% of pregnant women attended 4 or more ANC visits and 70% of the mothers did not receive all ANC services required. Also, 24.5% and 0.9% of pregnancies resulted in low birth weights and stillbirths, respectively. Normal birth weight is significantly associated with maternal religion, educational attainment, marital status, mothers who received long-lasting insecticide-treated nets, and those who had their urine tested. Inconsistency in logistics and medical supply to health facilities in the district should be addressed to ensure mothers who attend ANC clinics can receive all services required. Again, the rate of low-birth-weight children is very high. Therefore, there is a need for special attention from all stakeholders to reverse the trend.

### 5.1. Recommendations

The district health administration and other stakeholders in health should enhance education on the importance of antenatal care services including the necessity for early antenatal care and male involvement in women's health. The health authorities should institute supply-chain management systems to prevent logistics and medicine shortages. Again, there should be a study to ascertain the actual cause of the low birth weight in the district.

## Figures and Tables

**Table 1:** Background characteristics of participants.

Variable		Frequency	Percentage
Age group (years)	<20	41	17.6
	20-34	148	63.5
	≥35	44	18.9
Marital status	Legally married	215	92.3
	Cohabitation	18	7.7
Religion	Christianity	66	28.3
	Islam	167	71.7
Occupation	Unemployed	53	22.7
	Farmer	149	64.0
	Trader	31	13.3
Educational level	No education	113	48.5
	Primary/JHS	103	44.2
	SHS/tertiary	17	7.3
Parity	0-1	99	42.5
	2-3	78	33.5
	4+	56	24.0

**Table 2:** Antenatal services women received.

Variable		Frequency	Percentage
Number of ANC visits	0-1	12	5.2
	2-3	76	32.6
	4+	145	62.2
Gestational age determined	No	2	0.9
	Yes	231	99.1
Height taken	No	0	0.0
	Yes	233	100.0
PMTCT tested	No	3	1.3
	Yes	230	98.7
Iron-folic acid supplementation given	No	4	1.7
	Yes	229	98.3
Times folic acid received (*n* = 229)	1-2	45	19.6
	3-4	100	43.7
	5+	84	36.7
Hemoglobin level checked	No	11	4.7
	Yes	222	95.3
Urine tested	No	29	12.4
	Yes	204	87.6
Tetanus-toxoid vaccination received	No	31	13.3
	Yes	202	86.7
Weight taken	No	0	0.0
	Yes	233	100.0
SP tablets received	No	72	30.9
	Yes	161	69.1
Times SP tablets received (*n* = 161)	1	54	33.5
	2	107	66.5
Syphilis tested	No	5	2.1
	Yes	228	97.9
LLITN received	No	62	26.6
	Yes	171	73.4
Counselled on FP	No	10	4.3
	Yes	223	95.7
Counselled on nutrition	No	3	1.3
	Yes	230	98.7
Blood pressure checked	No	0	0.0
	Yes	233	100.0
Received minimum ANC service	No	163	70.0
	Yes	70	30.0

**Table 3:** Associations between background characteristics and a normal birth weight.

Variable	Birth weight		Pearson chi^2^		COR (95% CI); *p* value	AOR (95% CI); *p* value
	Normal (2.5-4.0 kg)	Abnormal (<2.5 kg)	Chi^2^	*p* value		
	*n* = 176 (%)	*n* = 57 (%)				
Age group (years)						
<20	33 (80.5)	8 (19.5)	1.160	0.560	1	
20-34	112 (75.7)	36 (24.3)			0.75 (0.32-1.78); 0.520	
≥35	31 (70.5)	13 (29.5)			0.58 (0.21-1.58); 0.287	
Marital status						
Cohabitation	10 (55.6)	8 (44.4)	4.215	0.040	1	1
Legal married	166 (77.2)	49 (22.8)			2.71 (1.01-7.24); 0.047	2.05 (1.33-6.89); 0.044
Religion						
Christianity	57 (86.4)	9 (13.6)	5.842	0.016	1	1
Islam	119 (71.3)	48 (28.7)			0.39 (0.18-0.85); 0.018	0.33 (0.08-0.39); 0.013
Occupation						
Farmer	115 (77.2)	34 (22.8)	0.671	0.715	1	
Trader	23 (74.2)	8 (25.8)			0.85 (0.35-2.07); 0.721	
Unemployed	38 (71.7)	15 (28.3)			0.75 (0.37-1.52); 0.425	
Educational level						
No education	84 (74.3)	29 (25.7)	11.919	0.003	1	1
Primary/JHS	82 (79.6)	21 (20.4)			1.35 (0.71-2.55); 0.359	1.20 (0.12-2.14); 0.876
SHS/tertiary	10 (58.8)	7 (41.2)			3.15 (0.04-0.61); 0.008	4.27 (0.08-0.88); 0.031
Parity						
0-1	78 (78.8)	21 (21.2)	1.6789	0.432	1	
2-3	55 (70.5)	23 (29.5)			0.64 (0.33-1.28); 0.208	
4+	43 (76.8)	13 (23.2)			0.89 (0.41-1.95); 0.772	

**Table 4:** Associations between antenatal services woman received and normal birth weight.

Variable	Birth weight		Pearson chi^2^		COR (95% CI); *p* value	AOR (95% CI); *p* value
	Normal (2.5-4.0 kg)	Abnormal (<2.5 kg)	Chi^2^	*p* value		
	*n* = 176 (%)	*n* = 57 (%)				
Number of ANC visits						
0-1	11 (91.7)	1 (8.3)	4.255	0.235	1	
2-3	61 (80.3)	15 (19.7)			0.31 (0.04-2.69); 0.288	
4+	104 (71.7)	41 (28.3)			0.23 (0.03-1.84); 0.167	

Gestational age determined						
Yes	176 (76.2)	55 (23.8)	3.101	0.078		
No	0 (0.0)	2 (100)			Omitted	

PMTCT tested						
Yes	176 (76.5)	54 (23.5)	3.214	0.073		
No	0 (0.0)	3 (100)			Omitted	

Iron-folic acid supplementation given						
Yes	172 (75.1)	57 (24.9)	1.318	0.251		
No	4 (100)	0 (0.0)			Omitted	

Times folic acid received (*n* = 229)						
1-2	39 (86.7)	6 (13.3)	3.209	0.201	1	
3-4	74 (74.0)	26 (26.0)			0.47 (0.18-1.26); 0.133	
5+	60 (71.4)	24 (28.6)			0.42 (0.16-1.12); 0.082	

Hemoglobin level checked						
No	4 (36.4)	7 (63.6)	9.587	0.002	1	1
Yes	172 (77.5)	50 (22.5)			6.02 (1.69–2.14); 0.006	0.15 (0.01-2.27); 0.17

Urine tested						
No	7 (24.1)	22 (75.9)	47.353	<0.001	1	1
Yes	169 (82.8)	35 (17.2)			5.18 (6.23-8.28); <0.001	6.59 (8.48-15.07); <0.001

Tetanus-toxoid vaccination received						
No	23 (74.2)	8 (25.8)	0.082	0.775	1	
Yes	153 (75.7)	49 (24.3)			1.14 (0.48-2.71); 0.775	

SP tablets received						
No	59 (81.9)	13 (18.1)	2.315	0.128	1	
Yes	117 (72.7)	44 (27.3)			0.59 (0.29-1.17); 0.131	

Times SP tablets received (*n* = 161)						
1	45 (83.3)	9 (16.7)	5.597	0.018	1	1
2	72 (66.7)	35 (33.3)			1.37 (0.16-0.86); 0.021	1.34 (0.10-1.12); 0.077

Syphilis tested						
No	1 (20.0)	4 (80.0)	8.529	0.003	1	1
Yes	175 (76.8)	53 (23.2)			3.21 (1.45-2.73); 0.022	1.27 (0.05-1.45); 0.885

LLITN received						
No	55 (88.7)	7 (11.3)	9.245	0.002	1	1
Yes	121 (70.8)	50 (29.2)			3.26 (0.11-0.65); 0.004	2.17 (0.03-0.92); 0.039

Counselled on FP						
No	4 (40.0)	6 (60.0)	7.140	0.008	1	1
Yes	172 (77.1)	51 (22.9)			5.06 (1.37-8.62); 0.015	3.13 (0.33-1.37); 0.331

Counselled on nutrition						
No	0 (0.0)	3 (100)	6.194	0.013		
Yes	176 (76.5)	54 (23.5)			Omitted	

Birth outcome						
Still birth	0 (0.0)	2 (100)	6.229	0.013		
Live birth	176 (76.2)	55 (23.8)			Omitted	

Received minimum ANC service						
No	116 (73.4)	42 (26.6)	2.391	0.122	1	
Yes	58 (82.9)	12 (17.1)			1.75 (0.86-3.58); 0.125	

## Data Availability

The data used to support the findings of this study can be made available from the corresponding author upon request.

## Abbreviations

ANC: Antenatal care
IPTp: Intermittent preventive treatment in pregnancy
HIV/AIDS: Human immunodeficiency virus/acquired immune deficiency syndrome
PMTCT: Prevention of mother-to-child transmission
WHO: World Health Organization
CHPS: Community-based health planning and services
EmONC: Basic emergency obstetrics and newborn care
SP: Sulphadoxine-pyrimethamine
LLITN: Long-lasting insecticide-treated net
JHS: Junior secondary school
SHS: Senior secondary school
FP: Family planning
COR: Crude odds ratio
AOR: Adjusted odds ratio.
